# Fabrication of Mechanically Reinforced Gelatin/Hydroxyapatite Bio-Composite Scaffolds by Core/Shell Nozzle Printing for Bone Tissue Engineering

**DOI:** 10.3390/ijms21093401

**Published:** 2020-05-11

**Authors:** Haeri Kim, Hanjun Hwangbo, YoungWon Koo, GeunHyung Kim

**Affiliations:** Department of Biomechatronic Engineering, College of Biotechnology and Bioengineering, Sungkyunkwan University, Suwon 16419, Korea; haeri1126@gmail.com (H.K.); hhwangbokorea@gmail.com (H.H.); ethenkoo@gmail.com (Y.K.)

**Keywords:** bioceramic, hydroxyapatite (HA), core-shell printing, hard tissue engineering

## Abstract

In tissue engineering, biocompatible scaffolds are used as 3D cell niches to provide a similar environment to that of native tissue for seeded cells to regenerate the target tissue. When engineering bone tissue, high mechanical strength and calcium phosphate composition are essential factors to consider. In this study, we fabricated biocompatible composite scaffolds composed of synthetic polymers (polycaprolactone (PCL) and poly (vinyl alcohol) (PVA)), natural polymers (gelatin and collagen) and bioceramic (hydroxyapatite; HA) for bone tissue engineering. The synthetic polymers were used to enhance the mechanical properties of the composite scaffolds while the natural protein-based polymers were used to enhance various cellular activities, such as cell adhesion and proliferation. Meanwhile, the bioceramic was introduced to promote osteogenic differentiation. Composite scaffolds were evaluated for their physical characteristics, such as mechanical, swelling and protein absorbing properties as well as biological properties (cell proliferation, alkaline phosphatase (ALP) activities and calcium deposition) with human osteoblast-like cells (MG63). Consequently, incorporation of hydroxyapatite into the gelatin/PVA (C-GPH) scaffold showed 5-fold and 1.5-fold increase in calcium deposition and ALP activities, respectively compared to gelatin/PVA scaffold (C-GP). Moreover, compressive modulus also increased 1.8-fold. Integration of PCL core into gelatin/PVA/hydroxyapatite scaffold (C-PGPH) further amplified the compressive modulus 1.5-fold. In conclusion, the scaffold that is reinforced with HA particles and integrated with PCL core of the struts showed significant potential in field of bone tissue engineering.

## 1. Introduction

Tissue engineering, which is achieved by integration of biological components such as cells and growth factors with biocompatible scaffolds, has the potential to regenerate damaged tissues. Three-dimensional (3D) scaffolds made from synthetic or natural biopolymers, which provide biophysical and biochemical support to cells, have been shown to be able to function as an artificial extracellular matrix (ECM), encouraging the formation of new tissues [1].

In general, biomaterials that closely resemble the intended tissue to be replaced are regarded as the most promising candidates for tissue engineering [2,3,4,5,6,7]. An ideal scaffold for bone engineering should be able to promote cell attachment/growth, promote osteoconduction and osteoinduction, have sufficient mechanical competence to sustain a mechanical load and be biodegradable to allow new tissue formation [8].

Natural polymers, such as alginate, cellulose, gelatin and collagen are widely used because of their ready availability and established biocompatibility, while synthetic polymers are renowned for low immunological effects [9]. Gelatin is relatively cheap and supports cell proliferation and differentiation; however, its poor mechanical properties have limited its use in hard tissue regeneration. Attempts have therefore been made to blend various synthetic polymers or bioceramics with gelatin to improve its mechanical strength. Synthetic polymer, poly(ε-caprolactone) (PCL), is a form of polyester that has been widely used for tissue engineering due to its relative resistance to chemical reactions and excellent mechanical properties [10]. Therefore, PCL has been applied to regenerate load-bearing tissues (cartilages and bones) of the human body.

Ceramic materials such as hydroxyapatite (HA) and tricalcium phosphate (TCP) are considered typical bioceramics because of their biocompatibility, osteoinductivity and osteoconductivity, making them ideal for scaffold fabrication for hard bone tissue engineering [11]. Although bioceramics have exceptional bioactive properties for bone tissue regeneration, their poor flexibility, high brittleness, and low processing ability have hindered the fabrication of precisely designed bioceramic scaffolds.

To overcome the shortcomings of natural polymers and bioceramics, biocomposite systems have been used in the fabrication of bioceramic-based scaffold. Current researches have shown numerous of fabrication strategies for temporary bone substitute scaffolds such as, solvent casting and lamination techniques [12], electrospinning [13,14] and extrusion manufacturing method [15]. However, most composite systems comprising bioceramics and biopolymers do not enhance in vitro cellular activities or improve mechanical properties. Some researchers have demonstrated that ceramic-based biocomposites increase osteogenic activities, while other researchers have reported that composite structures do not enhance biological activities, such as cell proliferation [16].

In our previous study, the mixture of gelatin and poly(vinyl alcohol) (PVA) has been used to produce a mechanically stable structure using a low temperature printing process and the weight fraction (5:5) of gelatin/PVA demonstrated suitable mechanical property without loss of cellular activities [17]. However, although the mixture can provide outstanding mechanical properties, poor biological activities of the scaffold can be one of impediments in hard tissue regeneration. To overcome low osteogenic activities, we used fibrillated collagen component that coated on the gelatin/PVA scaffold [18]. The collagen fibrillated scaffold resulted in enhancements in both cell growth and osteogenic differentiation. Nevertheless, the low mechanical strength and osteogenic activities of the scaffold has still limited its use on bone tissue engineering.

In this study, we developed an HA-based composite scaffold that promoted cellular activities and had good mechanical properties. In more detail, we incorporated HA into a gelatin/PVA mixture to enhance various cellular activities, including osteogenic responses. Furthermore, we utilized a core-shell nozzle to place PCL in the core region to improve the mechanical properties of the scaffold. Fabricated scaffolds were also coated with collagen solution and the collagen was fibrillated on the surface of the scaffold to increase cell attachment and proliferation [16]. We designed three types of scaffolds—(i) fibrillated-collagen-coated gelatin/PVA scaffolds (C-GP), (ii) fibrillated-collagen-coated gelatin/PVA/HA scaffolds (C-GPH) and (iii) fibrillated-collagen-coated core-shell scaffolds consisting of PCL (core) and gelatin/PVA/HA (shell) (C-PGPH). To appropriately fabricate these scaffolds, various material/processing parameters (HA weight concentrations, pneumatic pressures and print speeds) were varied. To determine the biological activities of the scaffolds, osteoblast-like-cells (MG-63 cells) were used. Integration of PCL into the core region of the ceramic-based scaffolds improved the mechanical properties of the composite scaffolds, while incorporation of HA into the scaffold substantially enhanced the osteogenic activities of the scaffolds.

## 2. Results

### 2.1. Scaffold Fabrication

**Figure 1:**
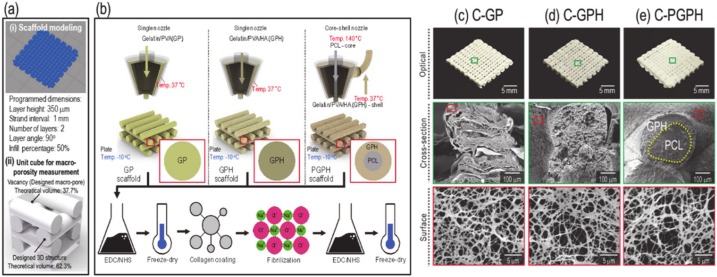
(**a**) Scaffold modeling-(i) and a schematic for macro-porosity calculation-(ii). (**b**) Schematic illustration of the fabrication of C-GP, C-GPH and C-PGPH scaffolds. Optical images and cross-sectional and surface scanning electron microscope (SEM) images of the fabricated scaffolds: (**c**) C-GP, (**d**) C-GPH and (**e**) C-PGPH.

Figure 1a showed the modeling of scaffolds manufactured by the 3D printer (i) and theoretical macro-porosity calculation (ii). Consequently, the theoretical macro-porosity was calculated to be 37.7%. Scaffolds C-GP, C-GPH and C-PGPH, shown in Figure 1b, were fabricated via a low temperature 3D printing process and the material/processing parameters used are described in Table 1. The same processing temperature used to fabricate scaffold C-GP was used to fabricate scaffolds C-GPH and C-PGPH. Moreover, the barrel temperature was set to 37 °C to facilitate easy flow of the gelatin/PVA/HA mixture in the nozzle. Then, lyophilized scaffolds were coated with collagen and the collagen was fibrillated with salt. Images of the fabricated composite scaffolds (C-GP, C-GPH and C-PGPH) with fibrillated collagen on their surfaces are shown in Figure 1c–e.

Each scaffold required the use of different printing parameters to achieve structural homogeneity due to differences in rheological properties. To determine the effect of the HA component on scaffold printing ability, material/printing parameters (HA weight fraction, pneumatic pressure and printing speed) were considered.

**Figure 2:**
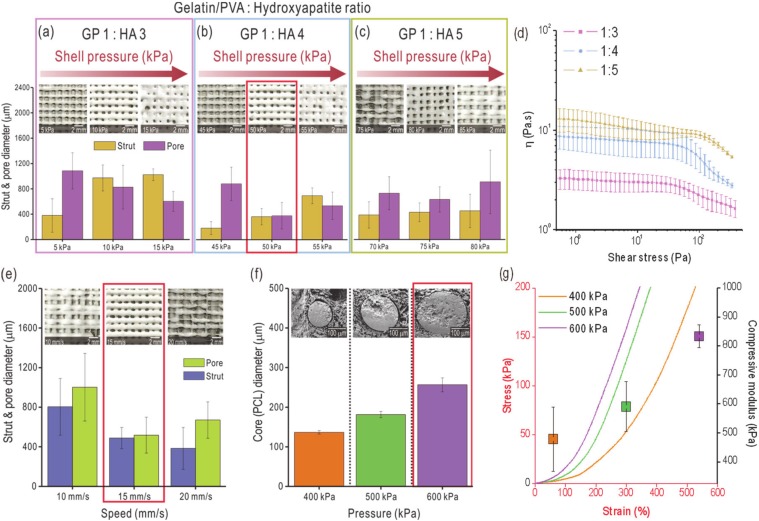
Printed strut and pore sizes for various pneumatic pressures and mixture ratios of gelatin/PVA (GP) and hydroxyapatite (HA) (**a**) 1:3, (**b**) 1:4 and (**c**) 1:5. (**d**) Complex viscosity (η) vs shear stress for various mixture ratios of GP and HA. (**e**) Strut and pore sizes for various nozzle moving speeds at a fixed mixture ratio (1:4) of GP and HA. (**f**) poly(ε-caprolactone) (PCL) diameter in the core region fabricated using a core/shell nozzle according to various pneumatic pressures applied in the core region and cross-sectional SEM images of the PCL-laden core regions. (**g**) Compressive stress-strain curves and moduli for various PCL diameters in the core region of the C-PGPH scaffold.

Figure 2a–c shows the strut and pore sizes for three HA weight fractions: 67 wt% (gelatin/PVA (GP) 1: HA 3), 80 wt% (GP 1: HA 4) and 90 wt% (GP 1: HA 5), for varied pneumatic pressures ranging from 5 to 80 kPa. In this test, a core/shell nozzle was used and PCL in the core region was extruded using a core temperature of 140 °C and pneumatic pressure of 600 kPa. Nozzle printing speed was set to 15 mm s^−1^. The higher the HA weight fraction, the higher the pneumatic pressure required to obtain a similar strut size due to higher viscosity, as shown in Figure 2d.

As shown in Figure 2a–c, the pneumatic pressure of 50 kPa for the 80 wt% HA mixture resulted in the most stable structure formation. Although 10 kPa for the 67 wt% mixture also resulted in stable structure formation, we selected the composite with the higher HA weight fraction because 80 wt% HA is more similar to the inorganic composition of human bone. After fixing the HA composition, we evaluated the effects of printing speeds ranging from 10 to 20 (mm s^−1^) on structure formation. As shown in Figure 2e, the printing speed of 15 mm s^−1^ was the most desirable as it produced a composite scaffold with an average strut and pore diameter of 443.9 and 472.4 µm, respectively.

After fixing the printing conditions (pneumatic pressure of 50 kPa and printing speed of 15 mm s^−1^) of the shell region to extrude the gelatin/PVA/HA, we measured the effect of pressure variations on the size of the PCL strut in the core region. Pneumatic pressures of 400, 500 and 600 kPa were investigated and, as expected, PCL diameter in the core increased as the pressure increased to 136.8, 181.5 and 256.6 µm, respectively (Figure 2f). As results of optimizing process parameters, the C-GP, C-GPH and C-PGPH scaffolds resulted in strut diameters of 424.3, 460.3 and 477.3 µm and pore sizes of 450.5, 448.2 and 450.5 µm.

To determine the effect of PCL diameter in the core region on the mechanical properties of the scaffolds, compressive stress-strain curves were generated for composite scaffolds with three different PCL diameters in the core region: 136.8, 181.5 and 256.6 µm. As shown in Figure 2g, compressive modulus increased gradually (478.8, 590.4 and 833.2 kPa for the three scaffolds, respectively) due to an increase in the PCL diameter (136.8, 181.5 and 256.6 µm, respectively). These results indicated that an increase in the PCL diameter in the core region directly enhanced the mechanical stability of the fabricated composite scaffolds.

### 2.2. Analysis of Pore Structures of the Composite Scaffolds

**Figure 3:**
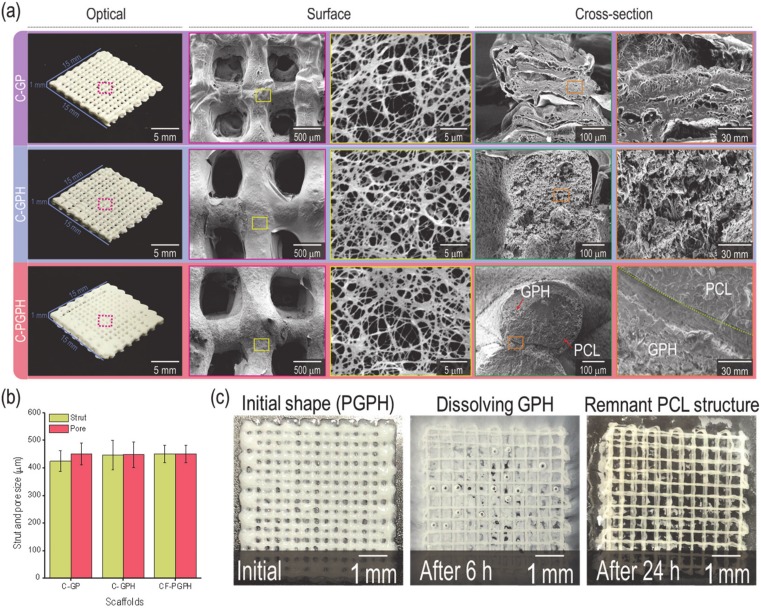
(**a**) Optical and SEM images of the fabricated scaffolds (C-GP, C-GPH and C-PGPH). (**b**) Measured strut and pore size of each scaffold. (**c**) Optical images showing strut changes after dissolving the GPH-laden shell region in 37 °C distilled water.

After fabricating core (PCL)/shell (gelatin/PVA/HA)-structured composite scaffolds using appropriate processing conditions and a core/shell nozzle, we coated the scaffolds with collagen solution to obtain fibrillated collagen. Optical images and surface/cross-sectional SEM images of collagen-coated gelatin/PVA (C-GP) and composite scaffolds [collagen-coated gelatin/PVA/HA (C-GPH) and collagen-coated scaffold composed of core (PCL)/shell (gelatin/PVA/HA) (C-PGPH)] are shown in Figure 3a. All scaffolds were fabricated to have the following dimensions: 15 × 15 × 1 mm. C-GP, C-GPH and C-PGPH scaffolds had strut diameters of 424.3, 460.3 and 477.3 µm and pore sizes of 450.5, 448.2 and 450.5 µm, respectively (Figure 3b and Table 2). Statistical analysis of sizes showed that geometrical differences between scaffolds were not statistically significant.

Magnified surface SEM images revealed that fibrillated collagen formed successfully on the surfaces of the scaffolds. The diameters of the C-GP, C-GPH and C-PGPH fibrillated collagen scaffolds were 210.1, 199.8 and 201.9 nm, respectively (Table 2).

### 2.3. FTIR and XRD

**Figure 4:**
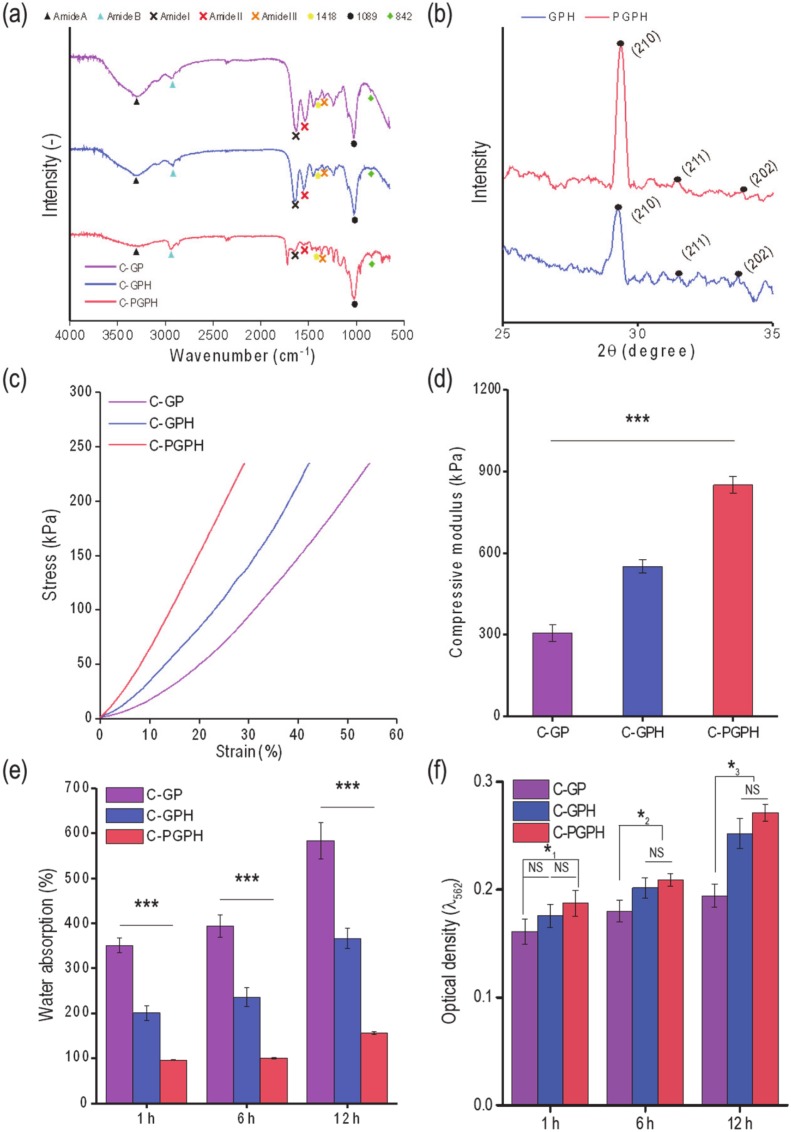
(**a**) Fourier-Transform Infrared (FTIR) spectra of scaffolds (C-GP, C-GPH and C-PGPH), (**b**) XRD results for C-GPH and C-PGPH scaffold. (**c**) Compressive stress-strain curves and (**d**) moduli of the scaffolds. (**e**) Water uptake ability and (**f**) protein absorption of the scaffolds at 1, 6 and 12 h. (NS > 0.05, *p* (*_1_) = 0.011, *p* (*_2_) = 0.006, *p* (*_3_) = 0.006 and *** *p* < 0.001).

Scaffolds (GP, GPH and PGPH) were analyzed using Fourier-Transform Infrared (FTIR) spectroscopy to detect material composition. PVA has FTIR spectra band characteristics at 1089 cm^−1^ and 842 cm^−1^, representing stretching vibrations of C-O and C-C and C-H bending, respectively. FTIR spectrometric characteristics of gelatin include peaks at 3276 cm^−1^ (amide A), 2937 cm^−1^ (amide B), 1652 cm^−1^ (amide I), 1552 cm^−1^ (amide II) and 1364 cm^−1^ (amide III). As shown in Figure 4a, all scaffolds exhibited these band characteristics, revealing that the scaffolds contained gelatin and PVA.

X-ray diffraction (XRD) was also used to verify the presence of HA in GPH and PGPH scaffolds (Figure 4b). Diffracted peaks were attributed to the HA crystal structure, indicating the presence of HA in these scaffolds [19,20,21].

### 2.4. Mechanical Properties

Compressive stress-strain curves are shown in Figure 4c; compressive moduli were calculated to be 0.31 ± 0.03, 0.55 ± 0.02 and 0.85 ± 0.03 MPa for C-GP, C-GPH and C-PGPH scaffolds, respectively (Figure 4d). The bioceramic in the scaffold (C-GPH) resulted in a 1.8-fold increase in compressive modulus compared to C-GP. Furthermore, integration of PCL core in the C-GPH scaffold (C-PGPH) resulted in 2.8-fold increase in compressive modulus compared to C-GP.

### 2.5. Water Uptake Ability and Protein Absorption

Structural sustainability of a scaffold can be evaluated by measuring its water uptake ability. To measure water absorption, scaffolds were immersed in distilled water and percentage weight change due to water absorption was measured. Figure 4e showed the water absorption (%) by the C-GP, C-GPH and C-PGPH scaffolds. After 12 h of immersing the scaffold in distilled water, C-GP showed greatest change in water absorption showing 584.0 ± 40.4% increase in weight. This was followed by C-GPH scaffold where it showed 366.2 ± 22.2% increase in weight, while C-PGPH scaffold showed minor change of 156.2 ± 3.7% increase in weight. Figure 4f shows the protein absorption of scaffolds at 1 h, 6 h and 12 h. While there was an overall increase in protein absorption, we observed substantially higher protein absorption in the HA-laden scaffolds (C-GPH and C-PGPH) than C-GP scaffold.

### 2.6. In Vitro Cellular Activities

**Figure 5:**
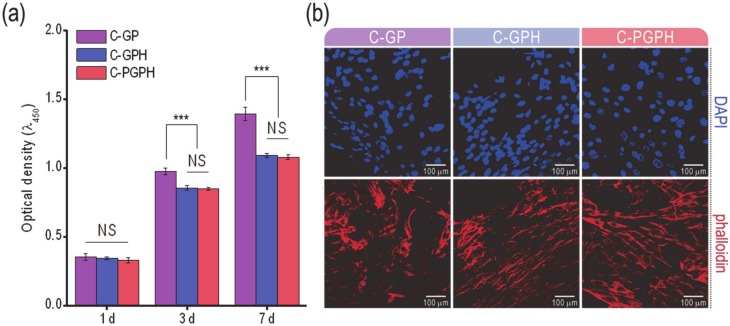
(**a**) Cell proliferation based on 3-(4,5-dimethylthiazol-2-yl)-2,5-diphenyl tetrazolium bromide (MTT) assay for cell cultured on scaffolds for 1, 3 and 7 days. (**b**) fluorescence images of the DAPI/phalloidin stained cells cultured on the scaffolds after 7 days. (NS means non-significance and *** *p* < 0.001).

As shown in 3-(4,5-dimethylthiazol-2-yl)-2,5-diphenyl tetrazolium bromide (MTT) results in Figure 5a, all scaffolds resulted in increase in cell proliferation from day 1 to day 7. However, C-GP scaffold resulted in higher cell proliferation after day 3, compared to HA-laden scaffolds. To observe the morphology of cells cultured for day 7, nuclear (blue)/cytoskeletal (red) staining of the scaffolds was evaluated and the fluorescent images are shown in Figure 5b. Cells cultured on C-GPH and C-PGPH scaffoldsformed filamentous (F)-Actin fibers and exhibited a stretched, flattened morphology.

**Figure 6:**
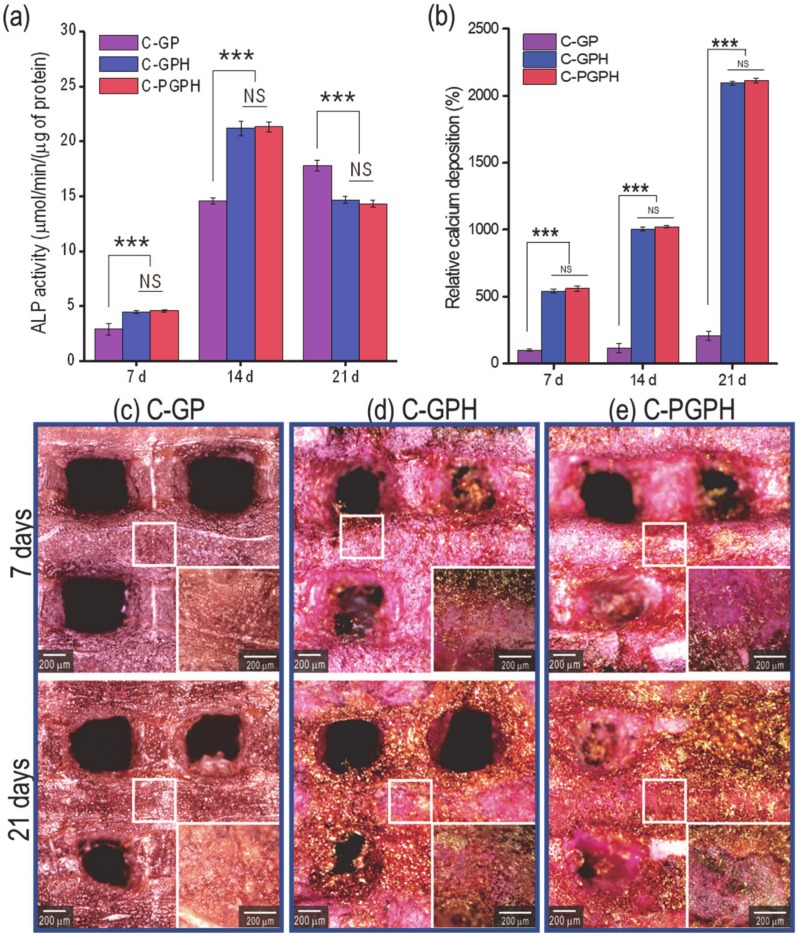
(**a**) Alkaline phosphatase (ALP) activity of cells at 7, 14 and 21 days, (**b**) Relative calcium deposition on scaffolds at 7, 14 and 21 days of culture and (**c**–**e**) optical images of scaffolds stained with alizarin red S. (NS means non-significance and *** *p* < 0.001).

ALP activity of each scaffold is shown in Figure 6a. The ALP activity of HA-laden scaffolds (C-GPH and C-PGPH) was 1.6-fold (4.5 ± 0.2 and 4.6 ± 0.1 µmol/min/(µg of protein), respectively) higher at day 7 and 1.5-fold (21.2 ± 0.7 and 21.3 ± 0.5 µmol/min/(µg of protein), respectively) higher at day 14 than the ALP activity of the C-GP scaffold (2.9 ± 0.5 (7 d) and 14.6 ± 0.3 µmol/min/(µg of protein) (14 d), respectively) on the corresponding days. Figure 6b shows relative calcium deposition on each scaffold at 7, 14 and 21 days as assessed by ARS staining (Figure 6c–e). To measure calcium deposition induced by osteoblast-like-cells, we calculated the difference between the total calcium content in the cultured scaffolds and the original calcium content in the scaffold. Relative to the calcium deposition of C-GP scaffold at day 7 (100 ± 10.1%), the results of calcium deposition of HA-incorporated scaffolds (C-GPH and C-PGPH) showed 545.6 ± 94.7% and 563.3 ± 63.3% at day 7, 1012.9 ± 132.6 and 1027.6 ± 92.1% at day 14 and 2108.2 ± 236.7% and 2126.6% at day 21, respectively. Meanwhile, C-GP scaffold only had increase of calcium deposition of 114.4 ± 26.8% and 206.7 ± 17.4% at days 14 and 21 respectively.

## 3. Discussion

3D scaffold fabrication is regarded as the principal approach for bone tissue engineering. Hence, there are active studies on novel fabrication methods. Fayyazbakhsh et al., has demonstrated fabrication of layered double hydroxides-hydroxyapatite/gelatin scaffolds for bone-tissue engineering via combining solvent casting and lamination techniques [12]. Furthermore, mineralization of electrospun nanofibers has also been promising method in fabrication of bioceramic-based scaffolds [13]. Wenz et al., successfully modified gelatin-based hydrogel with HA particles and fabricated a scaffold using an extrusion manufacturing method [15]. The ceramic-based scaffold had promising mechanical properties and promoted osteogenic differentiation. Hence, development of appropriate hydrogel is an essential field of study that has excellent printability and representative of native bone structure. In this study, we developed a novel hydrogel composed of gelatin/PVA/HA with integrated PCL core and, by using the hydrogel, a hybrid scaffold was fabricated via an extrusion-based printing process. The incorporation of HA particles into the scaffold exhibited a high degree of osteogenic differentiation with improved mechanical properties and morphological stability, while the integration of PCL core in the printed struts has significantly amplified mechanical properties and structural stability.

As shown in Figure 1b, representing the fabrication schematics of the C-GP, C-GPH and C-PGPH scaffolds, a single nozzle was utilized for C-GP and C-GPH scaffolds, whereas a core-shell nozzle for C-PGPH, which enabled integration of PCL core into C-GPH scaffold, was used. Furthermore, the low-temperature printing stage enabled the instant solidification of the hydrogel, allowing the stable mesh structure to be fabricated. However, the fabrication of C-PGPH scaffold was found to be problematic due to the printing temperature differences between core and shell region. Although the low temperature printing stage has resolved this temperature differential, the ensuing heat transfer from core to shell region have directed to distorted shell structure. This will be one of the overcoming issues in near future. Regarding the fabrication of C-PGPH scaffold, the core-shell nozzle with 860-µm shell diameter, which is much larger than the nozzle size (200 µm) of the single nozzle used for the fabrication of C-GP or C-GPH scaffold, was used, yet have produced similar strut diameter. This is because the ‘shell’ area of the core shell nozzle covers much larger area compared to the single nozzle while the pneumatic pressures used were similar (Table 2). Hence, this resulted in decrease in flow rate of GPH solution in the shell part of the core/shell nozzle, ensuing in similar strut diameter.

According to the rheological characterization of GPH (gelatin/PVA/hydroxyapatite) solution depicts inverse correlation between the weight fraction of hydroxyapatite and viscosity of the solution. This was apparent with the pneumatic pressures required to fabricate C-GPH scaffolds to desired strut and pore dimensions for the reasonings that the lower viscosity of GPH solution required lower shear stress applied by the pneumatic pressures to be ejected. Furthermore, 67 wt% HA mixture at 10 kPa could also produce stable structural formation, we have selected for 80 wt% HA in correspondence with the inorganic concentration found in human bone.

The desired strut diameter and pore size of the scaffolds were 500 µm. However, due to unforeseen circumstances such as shrinkage of the scaffolds, the actual strut diameter and pore size were lower compared to the designed dimension, even though freeze-drying process has mitigated the shrinkage to an extent. Moreover, the actual height of the scaffolds was measured to be lower compared to the designed scaffolds. This is due to the low viscosity of the hydrogel causing the layers to fuse. Furthermore, the macro-porosity of the programmed model was set as 37.7%. However, due to low viscosity nature of the GPH (gelatin/PVA/hydroxyapatite) solution, the struts between the layers fused together, causing the measured macro-porosity of the scaffolds to be lower.

The subsequent crosslinking via 1-Ethyl-3-(3-dimethylaminopropyl)carbodiimide (EDC)/N-hydroxysuccinimide (NHS) and ethanol solution allowed the fabricated scaffolds to retain their structure. These fabricated scaffolds were treated with collagen coating and fibrillization process with NaCl solution. The inorganic salt causes re-organization of collagen molecules, causing the formation of collagen fibrils [22]. From our previous study [18], we have already reported that providing topological cue through collagen fibrillization process enhanced the cell attachment as well as the cell proliferation and differentiation of the scaffold comprised of gelatin and PVA, so no additional evaluation of fibrillated collagen was performed in this study.

The diffracted peaks shown in XRD data attributed to HA, which indicates successful incorporation of HA in C-GPH and C-PGPH scaffolds (Figure 4b). The incorporation of HA particles into gelatin/PVA scaffold exhibited enhanced mechanical properties. HA nanoparticles enhances the effectiveness of load transfer from the polymer matrix to the nanoparticles resulting in better dispersion of load stress via interfacial adhesion between the polymer and nanoparticles, causing stiffer scaffold behavior [23]. The mechanical property of the scaffold was further enhanced with the integration of PCL core owing to the PCL that has relatively high stiffness. Although the mechanically improved scaffolds (C-PGPH and C-GPH) were inferior to native cortical bone tissue (compressive modulus of 14.1~27.6 MPa), it was higher than native trabecular bone (0.1~0.4 MPa) [24].

Water absorption of the scaffolds depends on hydrophilicity and micro-porosity and a respectable indicator of morphological sustainability [25,26]. Hydrophilic nature of gelatin and PVA component of C-GP scaffold resulted in highest water absorption, whereas incorporating of HA particles have resulted in the decrease in water absorption, indicating improved structural sustainability. The incorporation of HA has caused an increase in density leading to decrease in micro-porosity of the scaffold, resulting in significant decrease in water absorption. Meanwhile, the integration of PCL core in C-GPH struts displayed insignificant water absorption over time. This can be attributed to the impermeable nature of PCL core denoting only water absorption at shell region. In this study, the PCL region in core not only improved the mechanical properties of the scaffold but also their resistance to shape deformation by handling or shrinkage/swelling during the cell culture period compared to the other scaffolds.

Protein absorption, which is directly associated with topographical or chemical properties [27], is a key indicator of initial cellular activities, such as cell attachment and proliferation [28]. HA-incorporated scaffolds (C-GPH and C-PGPH) showed significantly higher protein absorption (Figure 4f). Hence, we have predicted high degrees of cell proliferation for HA-laden scaffolds (C-GPH and C-PGPH).

The cell proliferation of the scaffolds was assessed using the MTT assay (Figure 5a). Although there was insignificant difference of cell proliferation at day 1 between C-GP and HA-incorporated scaffolds, C-GP showed significantly higher cell proliferation after day 3. This phenomenon could be because the sporadically occurred differentiation in the HA-incorporated scaffolds can cause the inhibition of cell proliferation [29]. Similarly, it has been documented by Kim et al. that the incorporation of HA into poly(lactide-co-glycolide) composite scaffolds resulted in a decrease in cell proliferation [16]. Through this analysis, we can expect high levels of osteogenic differentiation for HA-laden scaffolds. This estimation could be supported by the morphological shape of the cultured cells at day 7 via DAPI/phalloidin staining in Figure 5b. In the images, the F-actin on the C-GPH and C-PGPH scaffolds was more elongated in shape compared to that of the C-GP scaffold. The spindle shapes of the cells can estimate a high degree of osteogenic differentiation credited to the synergistic effect of structural sustainability (significant low water uptake) reinforced by the HA and PCL structure and relatively high mechanical properties of the C-GPH and C-PGPH scaffolds.

Alkaline phosphatase (ALP) activity and calcium deposition are regarded as reliable indicators of osteogenic differentiation [30]. As shown in Figure 6a, ALP activity showed continual increase in C-GP scaffold from day 7 to 21, while HA-laden scaffolds showed the increase from day 7 to 14 (both significantly higher than C-GP scaffold) and decreased in day 21. This indicates that the HA component in the scaffolds promoted significantly higher ALP activity during initial cell culture. However, there was a decrease in ALP activity at day 21, indicating that the osteoblast-like-cells had differentiated [27,28,31]. Furthermore, although there was an overall increase in calcium deposition from day 7 to 21 in all the scaffolds, the presence of the HA particles appeared to promote osteogenic differentiation by encouraging osteoblasts to more actively deposit calcium into the scaffold. Although the C-PGPH scaffold showed superior mechanical properties to the C-GPH scaffold, calcium deposition in the C-GPH and C-PGPH scaffolds was similar. We are currently unable to exactly explain this phenomenon but we hypothesize that stiffness may affect the cell differentiation by serving as an indicator of the modulus of the scaffold surface. Furthermore, these results confirm the inverse relationship between cell proliferation and differentiation, coinciding with research conducted by Ruijtenberg et al. [26].

We attributed the significant increase of osteogenic differentiation to the synergistic effect of structural sustainability (significant low water uptake) reinforced by the HA and PCL structure and the relatively high mechanical properties of the C-GPH and C-PGPH scaffolds. Regarding future work, we will examine the osteogenic gene expressions via reverse transcription polymerase chain reaction (RT-PCR) and perform in vivo work.

## 4. Conclusions

In this study, we developed a HA/gelatin-based composite scaffold with outstanding cellular activities and mechanical properties by incorporating HA into a gelatin/PVA mixture and utilizing a core-shell nozzle to simultaneously print PCL in the core and HA/gelatin/PVA in the shell. To create a homogenous geometrical structure within the scaffolds, printing parameters (HA weight concentration, pneumatic pressure and printing speed) were optimized. Evaluation of mechanical properties revealed that integration of HA and PCL in the scaffold improved mechanical properties significantly, which is important for hard tissue engineering. Assessment of cellular activities such as ALP activity and calcium deposition revealed a notable enhancement in cell-mineralization in the gelatin/PVA scaffolds containing HA. To summarize, we successfully fabricated mechanically enhanced HA/gelatin-based composite scaffolds without loss of the biological activities of the scaffolds.

## 5. Materials and Methods

### 5.1. Materials

Poly(vinyl alcohol)(PVA) (Mw = 89,000–98,000 g/mol), gelatin from porcine skin (powder, high gel strength 280–302 g BloomAOAC, Type A) and poly(ε-caprolactone)(PCL) (Mw = ~14,000 g/mol) were purchased from Sigma-Aldrich (St. Louis, MO, USA). Type-I collagen from porcine skin was obtained from MSBio (Busan, Korea) and hydroxyapatite (HA) (particle size = 54.7 nm) was acquired from Sukgyung (Ansan, Korea).

### 5.2. Scaffold Fabrication

A modeling software (Simplify 3D version 3.1., simplify 3D Ltd., Ohio, USA) was used to programmed to be meshed structure of 15 × 15 × 1.4 mm consisting of 2 layers (A single layer consisting of two perpendicularly laid struts) of 1mm strut intervals and 700 μm layer height. The converted 3D model was printed using a 3D printer (Illuminaid, Seongnam, Korea). Through optimizing printing parameters (pneumatic pressure, moving speed), strut diameters were controlled.

Gelatin/PVA (GP) mixture was obtained by combining gelatin and PVA (0.5:0.5 wt%). Gelatin/PVA/HA (GPH) mixture was made by incorporating HA (4 wt%) into the gelatin/PVA (1 wt%) solution. GP and GPH structures were fabricated with a 200 µm single nozzle with the barrel temperature set to 37 °C and the working plate temperature set to −10 °C. The core (PCL)-shell (GPH) structure, which was designated ‘PGPH,’ was fabricated with a core-shell nozzle (core inner size: 260 µm and shell outer size: 860 µm). Processing temperatures of the shell barrel, core barrel and working stage were set to 37, 140 and −10 °C, respectively.

Gelatin component was cross-linked by immersing the scaffolds in 50 mM 1-ethyl-(3-3-dimethylaminopropyl) carbodiimide hydrochloride (EDC, Mw = 191.7 g/mol, Sigma-Aldrich, St. Louis, MO, USA) and 50 mM N-hydroxysuccinimide (NHS, Mw = 115.09 g/mol, Sigma-Aldrich, St. Louis, MO, USA) solution in 95% ethanol for 12 h at 4°C. Afterwards, the PVA was cross-linked by immersion in 95% ethanol for 12 h at 37 °C.

After lyophilizing the scaffolds, all scaffolds (GP, GPH and PGPH) were submerged in 0.2 wt% collagen solution for 30 min and then placed in 0.25 M NaCl at 37 °C for 1 h to induce fibrillation of the coated collagen. Coated collagen was also crosslinked with 50 mM 1-ethyl-(3-3-dimethylaminopropyl) carbodiimide hydrochloride (EDC, Mw = 191.7 g/mol, Sigma-Aldrich, St. Louis, MO, USA) solution in 95% ethanol for 30 min. Subsequently, the scaffolds were rinsed for 20 min with ethanol, phosphate-buffered saline (PBS) solution and distilled water, respectively. Finally, the scaffolds were freeze-dried. Collagen-coated scaffolds made using GP, GPH and PGPH were designated C-GP, C-GPH and C-PGPH, respectively.

### 5.3. Scaffold Characterization

Morphological structures of samples were observed under an optical microscope (model BXFM-32, Olympus, Japan) and scanning electron microscope (SEM; model SNE-3000M, SEC Inc., Suwon, Korea) and images were analyzed using ImageJ software version 1.51 (Image J; National Institutes of Health, Bethesda, MD, USA). The macro-porosity (%) of the model was calculated as (V_p_/V_T_ × 100), where V_p_ represents pore volume and V_T_ represents total vol1ume.

Gelatin/PVA/HA mixtures underwent rheological testing via a rotational rheometer (Bohlin Gemini HR Nano; Malvern Instruments, Malvern, UK) equipped with a cone-and-plate geometry (diameter: 40 mm; cone angle: 4°; gap: 150 μm). Shear stress sweeps were conducted by applying a range of shear stresses from 0.1 Pa to 100 Pa at a frequency of 1 Hz. All dynamic experiments were conducted at 37 °C.

Scaffolds were evaluated by FTIR analysis of FTIR spectra acquired using an FTIR spectrometer (model 6700, Nicolet, West Point, PA, USA) to detect and identify chemical groups specific to gelatin, PVA and collagen. FTIR spectra analyzed were the mean of 30 scans at 500–4000 cm^−1^ at a resolution of 8 cm^−1^.

Wide-angle X-ray diffraction (X’Pert PRO MRD; PANalytical, Malvern, UK) with CuKα radiation under beam conditions of 40 kV and 20 mA with spectrum collection at 2θ = 10 ~ 50° and a step size of 0.1° was performed to obtain the crystal peaks of HA.

### 5.4. Mechanical Properties

Mechanical properties of the scaffolds were assessed using a universal tensile testing machine (Top-tech 2000, Chemilab, Seoul, Korea) in compression mode. Samples sized 15 × 15 × 1 mm were freeze-dried. Five samples of each scaffold were tested. A 1 kgf load cell was used and the tests were conducted at a pressing speed of 0.05 mm s^−1^ at 25 °C without holding period.

### 5.5. Water Uptake Ability and Protein Absorption

Uptake of water by the scaffolds was assessed by weighing the scaffolds when dry and after immersion in distilled water for 1 h, 6 h and 12 h. Weight percentage increase due to water absorption was calculated as (W_h_ − W_0_)/W_0_ × 100, where W_h_ represents the weight of the scaffold after 1 h, 6 h and 12 h and W_0_ is the dry weight of the scaffolds.

A bicinchoninic acid (BCA) protein assay (Pierce Kit, Thermo Scientific, Waltham, MA, USA) was used to measure the protein absorption ability. The samples were immersed in 24-well plates with minimum essential medium (Life Science, St. Petersburg, FL, USA), supplemented with 10% fetal bovine serum (Gemini Bio-Products, Sacramento, CA, USA) and 1% antibiotic/antimycotic (Cellgro, Manassas, VA, USA) and incubated at 37 °C for 1, 6, 12 and 24 h. Subsequently, the scaffolds were washed with PBS three times and treated with 0.1% Triton X-100. Then, the lysate was added with 200 μL of BCA working reagent and then incubated for further 30 min at 37 °C. The protein concentration was determined from the absorbance at 562 nm using a plate reader (EL800, Bio-Tek Instruments, Winooski, VT, USA)

### 5.6. In Vitro Cell Culture

Scaffolds were sterilized via immersing the scaffolds in 70% ethanol solution for 2 h. Then, the scaffolds were washed with PBS solution followed by distilled water. This step was repeated 6 times. Afterwards, it was exposed to ultraviolet (UV) radiation for 24 h. Subsequently, MG-63 cells (CRL-1427, ATCC, Manassas, VA, USA) were seeded at a density of 1 × 10^5^ cells per sample. Minimum essential medium (Life Science) supplemented with 10% fetal bovine serum (Gemini Bio-Products) and 1% antibiotic/antimycotic (Cellgro) was provided to the seeded cells and the medium was changed every 2 days.

### 5.7. Cell Proliferation

To evaluate cell proliferation of the osteoblast-like cells, MTT assay (Cell Proliferation Kit I; Boehringer Mannheim, Mannheim, Germany) was performed after cell culture period of 1, 3 and 7 days. The osteoblast-like cells on the scaffold were incubated with 0.5 mg mL^−1^ MTT for 4 h at 37 °C. The OD was measured at 570 nm via microplate reader (EL800, Bio-Tek Instruments, VT, USA).

### 5.8. DAPI/Phalloidin Staining

After 1 and 7 days of cell culture, cells on the scaffolds were fixed in 3.7% formaldehyde solution to stain cell nuclei (diamidino-2-phenylindole (DAPI); Invitrogen, Carlsbad, CA, USA) and the actin cytoskeleton (Alexa Fluor 568 phalloidin; Invitrogen, Carlsbad, CA, USA). Stained cells were observed using confocal microscopy (LSM 700; Carl Zeiss, Germany).

### 5.9. ALP Activity and Alizarin Red S Staining

ALP activity was assayed by measuring the release of p-nitrophenol from p-nitrophenyl phosphate (pNPP). Cultured scaffolds must be preconditioned to acquire accurate ALP results. Cultured scaffolds were rinsed twice with PBS solution and equilibrated with ALP buffer (100 mM Tris-Cl, pH 9.5, 100 mM NaCl and 10 mM MgCl_2_). Then, samples were immersed in BCIP/NBT solution (Sigma, St. Louis, MO, USA) for 30 min. Enzymatic activity of the scaffolds were terminated by treatment of the scaffolds with 20 mM PBS solution containing EDTA. This was gently washed off with PBS solution and the scaffolds were then incubated in Tris buffer (10 mM, pH 7.5) containing 0.1% Triton X-100 surfactant for 10 min. Scaffolds were then assessed for ALP activity using an ALP kit (ALP-10, Sigma-Aldrich) and 100 µL of lysate was added to a 24-well tissue culture plate containing 100 µL pNPP solution. Stained samples were evaluated under an optical microscope and the quantity of pNPP transformed into p-nitrophenol and inorganic phosphate was measured by analyzing the absorbance at 405 nm using a microplate reader (Spectra III, SLT Lab Instruments, Salzburg, Austria).

Mineralization of osteoblastic cells was quantified and evaluated via alizarin red S (ARS) staining in 24-well plates. Samples were washed three times with PBS solution and immersed in 70% ethanol at 4 °C for 1 h. After samples were air-dried, they were stained with 40 mM ARS solution (pH 4.2) for 1 h and rinsed with distilled water. Samples were de-stained via treatment with 10% cetylpyridinium chloride in 10 mM sodium phosphate buffer (pH 7.0) for 15 min. Samples were evaluated under an optical microscope and optical density was measured at 562 nm using a microplate reader. ALP activity and calcium deposition were normalized to the total protein content. All values are reported as means ± SDs (*n* = 5).

### 5.10. Statistical Analyses

Statistical analyses were conducted using SPSS software version 12.0 (SPSS Inc., Chicago, IL, USA). Single-factor analysis of variance, LSD test was used and no significant differences are depicted as NS, where *p* > 0.05. Significant difference is represented as * *p* < 0.05 and *** *p* < 0.001.

## Figures and Tables

**Table 1:** Processing parameters for scaffold fabrication.

Scaffold	Working Plate Temp. (°C)	Barrel Temp. (°C)	Core temp. (°C)	Moving Speed (mm/s)	Pressure in Core (kPa)	Pressure in Shell (kPa)	Gelatin (wt%)	PVA (wt%)	HA (wt%)
C-GP	–10	37	-	15	40		0.5	0.5	-
C-GPH			-		50				4
C-PGPH			140		600	50			

**Table 2:** Strut diameter, pore size, macro-porosity, collagen fibril size, PCL diameter in the core region and shell thickness of the fabricated scaffolds.

Scaffold	Strut Diameter (μm)	Pore Size (μm)	Macro-Porosity (%)	Fibril Diameter (nm)	PCL Diameter (μm)
Designed dimensions	500	500	37.7	-	-
C-GP	424 ± 37	450 ± 39	30.28 ± 1.84	210 ± 37	-
C-GPH	460 ± 45	448 ± 46	28.09 ± 2.14	199 ± 40	-
C-PGPH	477 ± 55	450 ± 32	25.31 ± 1.71	201 ± 38	212 ± 51

## Abbreviations

ALP	Alkaline phosphatase
ARS	Alizarin red S
BCA	Bicinchoninic acid
C-GP	Fibrillated-collagen-coated gelatin/PVA scaffolds
C-GPH	Fibrillated-collagen-coated gelatin/PVA/HA scaffolds
C-PGPH	Fibrillated-collagen-coated core-shell scaffolds consisting of PCL (core) and gelatin/PVA/HA (shell)
DAPI	Diamidino-2-phenylindole
EDC	1-ethyl-(3-3-dimethylaminopropyl) carbodiimide hydrochloride
FTIR	Fourier-transform infrared
HA	Hydroxyapatite
NHS	N-hydroxysuccinimide
PCL	Poly(ε-caprolactone)
PVA	Poly(vinyl alcohol)
SEM	Scanning electron microscope
XRD	X-ray diffraction
